# Quercetin ameliorates testosterone secretion disorder by inhibiting endoplasmic reticulum stress through the miR-1306-5p/HSD17B7 axis in diabetic rats

**DOI:** 10.17305/bjbms.2021.6299

**Published:** 2021-09-28

**Authors:** Di Wang, Yan Li, Qian-qian Zhai, Yun-feng Zhu, Bei-yan Liu, Yun Xu

**Affiliations:** Department of Endocrinology, The First Affiliated Hospital of Xinxiang Medical College, Xinxiang, China

**Keywords:** Quercetin, endoplasmic reticulum stress, miR-1306-5p, HSD17B7, Janus kinase 2/signal transducer and activator of transcription-3 axis

## Abstract

Testicular damage and testosterone secretion disorder are associated with diabetes mellitus. Quercetin, a common flavonoid, has antioxidant, anti-cancer, and blood sugar lowering effects. Therefore, this study aims to investigate the effect of quercetin on the reproductive system of male rats with diabetes *in vivo* and *in vitro* and elucidate its mechanism. Streptozotocin (STZ) induction was used to establish a diabetes model in 40 male Sprague Dawley rats, which were subsequently administered with 20 or 50 mg/kg of quercetin. Leydig cells of rat testes were treated by high glucose (HG) followed by 5 or 10 μM quercetin. Two doses of quercetin increased rat body weight and testicular weight, decreased blood glucose, and inhibited oxidative stress. Quantitative real-time polymerase chain reaction and Western blotting revealed that quercetin alleviated STZ-induced testicular damage and promoted testosterone synthesis. Both doses of quercetin reduced reactive oxygen species and malondialdehyde levels, and increased superoxide dismutase level in HG-treated cells. Both, *in vivo* and *in vitro* results confirmed that a high dose of quercetin was more effective. MiR-1306-5p was upregulated in testicular tissue of diabetic rats and HG-treated cells. 17β-hydroxysteroid dehydrogenase (HSD17B7) was a target of miR-1306-5p and HSD17B7 was downregulated in STZ-induced rat tissues and HG-treated cells. HSD17B7 overexpression reversed the increase of C/EBP homologous protein (CHOP) and glucose-regulated protein 78 (Grp78) protein levels as well as eIF2α phosphorylation level and promotion of cell apoptosis caused by miR-1306-5p overexpression. Moreover, overexpression of HSD17B7 activated the Janus kinase 2/signal transducer and activator of transcription 3 axis in HG-treated cells. In conclusion, quercetin inhibits endoplasmic reticulum stress and improves testosterone secretion disorder through the miR-1306-5p/HSD17B7 axis in diabetic rats.

## INTRODUCTION

Diabetes mellitus (DM) is a metabolic disease caused by glucose metabolism disorders. DM affects male reproductive function in many aspects, including spermatogenesis, testosterone secretion, ejaculatory dysfunction, and so on [1]. The relative weights of the testis, epididymis, seminal vesicles, and prostate of diabetic rats were significantly reduced. The daily sperm production, epididymal sperm count, sperm motility, live sperm, serum testosterone secretion levels, and testicular 3β- and 17β-hydroxysteroid dehydrogenase (HSD17B7) activities were also significantly reduced [2]. The body weight, blood sugar, and insulin levels of type 2 diabetic mice were increased, sperm production and steroid production were decreased, the production of antioxidant enzymes, and the disorder of lipid distribution [3]. The levels of testosterone secretion, follicle-stimulating hormone and luteinizing hormone (LH) in diabetic rats were decreased significantly [4]. The previous studies reported that DM had a negative impact on the reproductive efficiency of male offspring after puberty and the balance of testicular tissue oxidation/antioxidation [5,6]. Men with low levels of testosterone secretion could increase their risk of developing diabetes in the future [7].

Quercetin, as a common flavonoid, is widely distributed in fruits and vegetables [8]. As an effective antioxidant, it has many beneficial effects, such as anti-cancer [9], anti-inflammatory, antioxidant [10], and hypoglycemic properties [11]. A previous study revealed that quercetin could prevent endoplasmic reticulum (ER) stress and rebuild ER homeostasis by reducing endothelial cell apoptosis and increasing antioxidant status [12]. Quercetin treatment improved the reduction of seminiferous tubule diameter, biochemical parameters (malondialdehyde [MDA], superoxide dismutase (SOD), glutathione peroxidase, and serum testosterone level), and germ cell apoptosis, suggesting that quercetin treatment could protect testis from the toxic effect of cadmium [13]. In addition, a study revealed that docetaxel-treated rats significantly reduced sperm motility, sperm concentration, testicular and epididymal weight, and increased abnormal sperm rate and histopathological changes, while quercetin treatment could eliminate the effect of docetaxel [14]. Sitagliptin was a hypoglycemic agent approved for the treatment of type 2 diabetes by inhibiting the dipeptidyl peptidase IV enzyme [11]. Sitagliptin was used to alleviate reproductive dysfunction in Sprague-Dawley male rats [15]. In addition, it was reported that sitagliptin treatment significantly increased serum testosterone levels and lowered cholesterol levels in renal ischemia/reperfusion rats [16].

The imbalance between protein load and the folding capacity of the ER causes ER stress, resulting in the accumulation of unfolded or misfolded proteins in the ER cavity. ER stress or ER homeostasis damage was closely related to the pathology of reproductive diseases [17,18]. ER stress caused testicular damage and disorder of testosterone secretion by inducing the apoptosis of testicular Leydig cells in rats [19,20]. Excessive production of reactive oxygen species (ROS) had pathological effects on male sperm, in addition to DNA damage; it would also reduce vitality, motility and morphological changes, which might lead to caspase activation and cell apoptosis [21]. A study revealed that oral betaine in diabetic mice could significantly improve the weight of reproductive organs and testicular morphology. In addition, the levels of reactive oxygen species and malondialdehyde in testicular tissues were significantly reduced [22]. Quercetin improved polycystic ovary syndrome in women of reproductive-age by reducing testosterone secretion, LH and insulin resistance. However, there were not largely studies on quercetin in the treatment of testosterone secretion disorders in men or male animals [23]. A previous study reported that quercetin mediates ER stress induced protective autophagy and apoptosis in ovarian cancer through the p- signal transducer and activator of transcription (STAT)3/Bcl-2 axis [24]. In addition, quercetin reduced apoptosis and inflammatory response of vascular endothelial cells cultured with high concentration of glucosamine through the ER stress pathway [25], and it improved diabetic encephalopathy in db/db mice through the SIRT1/ER stress pathway [26]. Although quercetin could relieve diabetic syndrome, its specific mechanism was still ambiguous in the treatment of abnormal secretion of testosterone in rats caused by diabetes.

HSD17B7 is a multifunctional protein in mammals, belonging to the HSD17B7 family and the squalene post cholesterol biosynthetic enzyme family involved in steroid hormone metabolism. HSD17B7 has not been largely reported in various diseases. HSD17B7 participated in the cholesterol synthesis pathway during the conversion of lanosterol to sterol [27]. Studies have shown that high triglycerides and low high-density lipoprotein cholesterol levels are markers of high risk of type 2 diabetes [28,29]. Type 2 DM (T2DM) led to glucose and lipid metabolism disorder and insulin resistance, resulting in elevated blood glucose and weight loss, resulting in adverse reactions in T2DM patients. A previous study showed that glyphosate interfered with the expression of StAR and CYP17A1, and inhibited the synthesis and secretion of testosterone through the activation of the PERK/eIF2α signaling pathway in Leydig cells mediated by ER stress [30]. HSD17B7 was mainly distributed on the ER membrane; hence, we might suspect that it might be involved in ER stress in regulating testicular secretion in diabetic rats.

The study aimed to investigate the effect of quercetin on the imbalance of testosterone secretion in Streptozotocin (STZ)-induced diabetic rats and its mechanism. Moreover, we also determined the effect of quercetin on HG-treated testosterone secretion levels in Leydig cells. An in-depth understanding of the protective effect of quercetin and its mechanism of HG-induced ER stress in mesenchymal cells might help design new therapies for the treatment of male endocrine disorders caused by diabetes.

## MATERIALS AND METHODS

### Animal model

Forty 6-week-old male Sprague Dawley rats (weighing 160-180 g) were provided by the animal center of the first affiliated hospital of Xinxiang medical college, China. Rats were injected intraperitoneally with 60 mg/kg (STZ, Sigma-Aldrich Corporation, St. Louis, Mo, USA) [31], followed by 60 mg/kg for another 2 consecutive days, and the random blood glucose concentration in tail vein was no less than 16.67 mmol/L for two times. According to the guidelines of the first affiliated hospital of Xinxiang Medical College, the animals were housed in a 12 hours light/dark cycle at 21 ± 3°C, and relative humidity 30-70%. The research was approved by the Institutional Animal Ethical Committee of the first affiliated hospital of Xinxiang Medical College (XXYXY-2019-045). Throughout the experiment, animals had free access to food and water.

### Animal grouping and treatment

The rats were divided into five groups (n = 8 per group): (1) control; (2) STZ-induced diabetic rats; (3) diabetic rats administered with sitagliptin (70 mg/kg BW) [11]; (4) diabetic rats administered with quercetin (20 mg/kg BW); and (5) diabetic rats administered with quercetin (50 mg/kg BW) [32,33]. Sitagliptin was reported to reduce the ER stress in diabetic rats [34]. Quercetin and sitagliptin were intraperitoneally injected into rats for 6 weeks, and rats in the control group were injected with the same amount of normal saline. The Accu-Chek blood glucose meter (Roche Diagnostics, Indianapolis, USA) regularly recorded the fasting blood glucose of laboratory animals. Body weight and testicular weight were measured in rats with different treatment using electronic balance (UW2200H, Shanghai Shengke Instrument Equipment Co., Ltd, Shanghai, China.). After the experimental period, animals were fasted overnight and euthanized by excess pentobarbital sodium, and testicular samples were collected and stored for further analysis.

### Cell culture and cell treatment

Leydig cells of rat testis (Cat.# FE1489) were purchased from American Type Culture Collection (ATCC, Manassas, VA, USA), cultured in Dulbecco’s modified Eagle’s medium with 10% fetal bovine serum (FBS, Gibco, Grand Island, NY, USA). Cells were cultured in a humidified incubator with 5% CO_2_ at 37°C. Leydig cells were treated with HG (46.2 mmol/L) to simulate type 1 DM cells, and 2.8 mmol/L glucose was used as a control. Leydig cells were treated with HG (46.2 mmol/L) alone or together with sitagliptin (20 μM) or quercetin (5 or 10 mM) [25].

MiR-1306-5p mimic, NC mimic (a negative control of miR-1306-5p mimic), pcDNA-HSD17B7 (HSD17B7), and pcDNA3.1 (Vector, a negative control of HSD17B7) were purchased from GenePharma Co. (Shanghai, China), and were transfected into cells with Lipofectamine™ 3000 (Invitrogen, Carlsbad, CA, USA). The pcDNA-HSD17B7 was used to overexpress the expression of HSD17B7, and miR-1306-5p mimic was used to upregulate the miR-1306-5p level.

### Detection of cell viability

The cells were inoculated in 96-well plates (Corning, NY, USA) and cultured in 5% CO_2_ at 37°C, and then treated with different doses (0, 2.5, 5, 10, 20, 40, 80, or 160 μM) of quercetin. Subsequently, cell proliferation activity was detected with a Cell Counting Kit (CCK-8) assay (Dojindo Laboratories, Kumamoto, Japan). After incubating for 24 or 72 hour, 10 μL of CCK-8 was added to each well, and then incubated for 1 hour at 37°C. A microplate reader was used to measure the absorbance at 450 nm.

### RNA extraction and Quantitative real-time polymerase chain reaction (RT-qPCR)

The TRIzol reagent (Invitrogen, Carlsbad, CA, USA) was used to extract total RNA from cells and tissues, according to the manufacturer’s instructions. The SuperScript III First-Strand Synthesis System (Thermo Fisher, Waltham, MA, USA) was used to reverse-transcription of miR-1306-5p into cDNA. Next, we used the commercial kit SYBR® Premix Dimer Eraser kit (Takara, Dalian, China) to identify gene expression, according to the manufacturer’s instructions, U6 or GAPDH was used as a control. RT-qPCR was measured with fluorescence quantitative PCR (ABI 7900 HT, ABI, USA). 20 μL of reaction system was used as below: SYBR Premix Ex Taq II (2 ×) 10.0 μL, forward primer (10 μmol/L) 0.8 μL, reverse primer (10 μmol/L) 0.8 μL, cDNA template 2.0 μL, dH_2_O (RNase free) 6.4 μL. PCR conditions were as below: 95°C for 1 minute, 56°C for 10 second, and 72°C for 15 second for 35 cycles. The melting curve confirmed the specificity of PCR products. Primers sequences are as follows: P450scc forward: 5¢-TTC CCA TGC TCA ACA TGC CTC-3¢, reverse: 5¢- ACT GAA AAT CAC ATC CCA GGC AG-3¢; 17β-HSD forward: 5¢-ATG AGC CCG TTT GCC TCT G-3¢, reverse: 5¢-CCA CAG GTA ACA AGT CTT GGT C-3¢; eNOS forward: 5¢-GAC GCT ACG AGG AGT GGA AG, reverse: 5¢- CCT GTA TGC CAG CAC AGC TA; calcitonin gene-related peptide (CGRP) forward: 5¢-ACT TGA ACG CCA TCA CCT AC-3¢ reverse: 5¢-GTC AGC TTG TGG CTC TTC AT-3¢; iNOS forward: 5¢-GCT ACA CTT CCA ACG CAA CA-3¢ reverse: 5¢-ACA ATC CAC AAC TCG CTC CA-3¢; endothelin-1 (ET-1) forward: 5¢-CGC TGG TAG CAA GTG ATT-3¢ reverse: 5¢-CTT TCC CTG AAA TGT GCC-3¢; C/EBP homologous protein (CHOP) forward: 5¢-CTT CAC TAC TCT TGA CCC TG-3¢ reverse: 5¢-CAT TCT CCT GCT CCT TCT C-3¢; glucose regulated protein of 78 kDa (Grp78) forward: 5¢-TCA GCC CAC CGT AAC AAT CAA G-3¢ reverse: 5¢-TCC AGT CAG ATC AA ATG TAC CCA GA-3¢; miR-1306-5p forward: 5¢-GGC AGA GGA GGG CTG TTC-3¢ and reverse, 5¢-GTG CGT GTC GTG GAG TCG-3; U6 forward: 5¢-CTC GCT TCG GCA GCA CA-3¢ and reverse, 5¢-AAC GCT TCA CGA ATT TGC GT-3¢; StAR forward: 5¢-CCT GAG CAA AGC GGT GT-3¢, reverse: 5¢-TGA TGA TGG TCT TTG GCA GC-3¢; 3β-HSD forward: 5¢- AAC TGC CAC TTG GTC ACA CTG TC-3¢; reverse: 5¢-GTC CCG ATC CAC TCC GAG GTT T-3¢; GAPDH forward: 5¢-GGT GGT CTC CTC TGA CTT CAA CA-3¢ and reverse, 5¢-GTT GCT GTA GCC AAA TTC GTT GT-3¢. Fold change was calculated by the 2^−DDCt^ method.

### Western blot assay

The proteins were extracted from cells using radio-immunoprecipitation assay lysis buffer (Beyotime, Shanghai, China). The total protein concentrations were detected with bicinchoninic acid protein assay kit (Beyotime, Shanghai, China). The same amount of proteins (20 µg) was packed into each electrophoresis tank, then separated by 12% sodium dodecyl sulfate-polyacrylamide gel electrophoresis, and transferred to polyvinylidene fluoride membranes (Millipore, Billerica, MA). Subsequently, the membranes were blocked with 10% skim milk at approximately 25°C. Then, the membranes were washed and incubated with the primary antibodies at 4°C for about 24 hour. Primary antibodies including rabbit anti-CHOP (ab194533, 1:400, Abcam, Cambridge, UK), rabbit anti-Grp78 (ab21685, 1: 500, Abcam), rabbit anti-eIF2α (ab169528, 1: 400, Abcam), rabbit anti-cleaved caspase-3 (ab32042, 1: 400, Abcam), rabbit anti- Janus kinase (JAK)2 (ab108596, 1: 300, Abcam), and rabbit anti-STAT3 (ab68153, 1: 500, Abcam) were used for incubated with goat anti-rabbit IgG H&L (HRP) (ab205718, 1:1000, Abcam) for 1 hour at room temperature. Finally, they were put into an enhanced chemiluminescence system (Bestbio, Shanghai, China) to develop the signals and scan the protein bands with Image J software. GAPDH was used as an endogenous control.

### Determination of ROS in cells and tissues

2’-7’-Dichlorodihydrofluorescein diacetate was used to detect the production of ROS in cells and tissues. Cells and tissues were oxidized to fluorescence in the presence of peroxide 2’,7 -Dichlorofluorescein (DCF). The cells were separated by trypsin EDTA, centrifuged, washed with phosphate buffer saline (PBS), and treated with 10 μM H2DCF-DA at 37°C for 30 minutes. Subsequently, the cells were washed twice with PBS and treated with 5 or 10 μM quercetin in a CO_2_ incubator at 37°C for 1 hour. The cells treated with hydrogen peroxide used as a positive control. The treated cells were washed with PBS again, and then the fluorescence DCF intensity was measured with FACSCalibur flow cytometer (BD Bioscience, California, USA).

### Enzyme-linked immunosorbent assay (ELISA)

MDA level and SOD activity in testicular tissues were detected by ELISA. After transfection, the cells were collected and centrifuged. According to the manufacturer’s instructions, MDA level and SOD activity in the cell supernatant were determined with ELISA kits (Thermo Fisher Scientific, Waltham, MA, USA). Testosterone secretion in serum and testis was also detected by an ELISA kit (Thermo Fisher Scientific, Waltham, MA, USA).

### Luciferase reporter gene assay

The potential binding sites between miR-1306-5p and HSD17B7 were predicted with Starbase (http://starbase.sysu.edu.cn/index.php). Luciferase reporter gene assay confirmed this assumption. The wild type or muted 3’UTRs of HSD17B7 was inserted into the pGL3-reporter plasmid (Promega, Madison, WI, USA), and the luciferase reporter plasmids HSD17B7 3’UTRs-WT and HSD17B7 3’UTRs were constructed. Next, the HSD17B7 3’UTRs or HSD17B7 3’UTRs-WT and miR-1306-5p mimic or a corresponding negative control (NC mimic) were co-transfected into HEK-293T cells with Lipofectamine™ 3000 (Invitrogen, Carlsbad, CA, USA) when grown to approximately 70% confluence. After transfection for 24 hours, we harvested the cells and analyzed them with a dual luciferase reporter kit (Promega, Madison, Wisconsin, USA) to standardize luciferase reporter activity to Renilla luciferase activity.

### Cell apoptosis assay

Flow cytometry with Annexin V/propidium iodide (PI) staining was used to measure the apoptosis of Leydig cells. First, the collected cells were centrifuged at 1000 rpm for 10 minutes. Then, the culture medium was discarded, and the cells were made into a single cell suspension with a density of 1×10^6^ cells/mL. 10 µg/mL Annexin V-FITC was added into cell suspension, incubated in the dark for 10 minutes, and then 20 µg/mL PI was added. The apoptosis of Leydig cells was detected with Flow cytometry.

### Ethical statement

These animal surgeries were carried out in accordance the National Institutes of Health on Animal Care Guidelines. This study was approved by the First Affiliated Hospital of Xinxiang Medical College.

### Statistical analysis

SPSS version 20 (SPSS Inc., Chicago, IL, USA) was used to analyze the data, and all data were expressed as mean ± SEM. Student’s *t*-test was used for the two groups, and analysis of variance (ANOVA) was used to analyze the statistical significance of multiple groups, and the Tukey-Kramer correction was used for multiple comparisons. *p* < 0.05 was considered statistically significant.

## RESULTS

### Quercetin increased body weight, testicular weight, and testosterone secretion in STZ-induced rats

To examine the effect of quercetin on the disorder of hormone secretion, two doses of quercetin were used to treat the diabetic rats, and sitagliptin was used as a positive drug.

**FIGURE 1:**
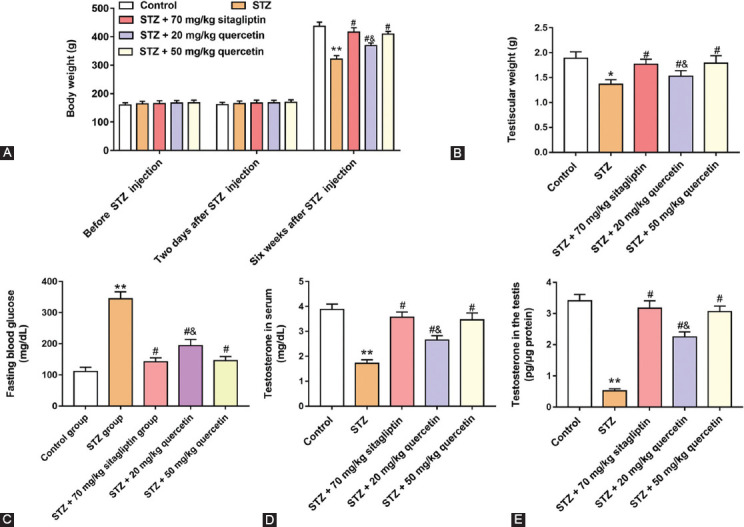
Effects of quercetin on body weight, testicular weight, blood glucose content, and testosterone secretion in Streptozotocin (STZ)-induced rats. Rats were administered with STZ, 70 mg/kg sitagliptin (a positive drug), and two doses (20 or 50 mg/kg) of quercetin. Administration of STZ into rats was to induce diabetes. (A) Rat body weight was measured in five groups (n = 8 per group) before STZ injection, two days after STZ injection and six weeks after STZ injection. Testicular weight (B) and fasting blood glucose (C) were detected in rats. Testosterone secretion in serum and testis was detected in rats. **P < 0.01, compared with control group; ^#^*P <* 0.05, compared with STZ group; ^&^*P <* 0.01, compared with STZ + 70 mg/kg sitagliptin or STZ + 50 mg/kg quercetin group.

Compared with rats in the control group, rats in other groups did not change their body weight before STZ injection and 2 days after STZ injection (*p* > 0.05) (Figure 1A). Six weeks after administration of STZ, diabetic rats reduced body weight, and two doses (20 or 50 mg/kg) of quercetin treatments increased diabetic rat body weight (*p* < 0.05). To observe the effects of the DM on testicular development, testicular weight of rats was examined. As shown in Figure 1B, compared with the control group, rats administered with STZ decreased the testicular weight, and quercetin treatments eliminated the effect of diabetes on testicular weight (*p* < 0.05). In addition, rats administered with STZ also increased the contents of fasting blood glucose and decreased testosterone secretion in serum and testis, compared with control group, and quercetin treatments lowered blood sugar, and increased testosterone secretion (*p* < 0.05) (Figure 1C-E). These results indicated that the effect of the high dose was better than that of the low dose. Together, these data suggested that quercetin may help to alleviate the disorder of testosterone secretion caused by diabetes.

### Quercetin attenuated oxidative stress in diabetic rats and HG-treated Leydig cells

**FIGURE 2:**
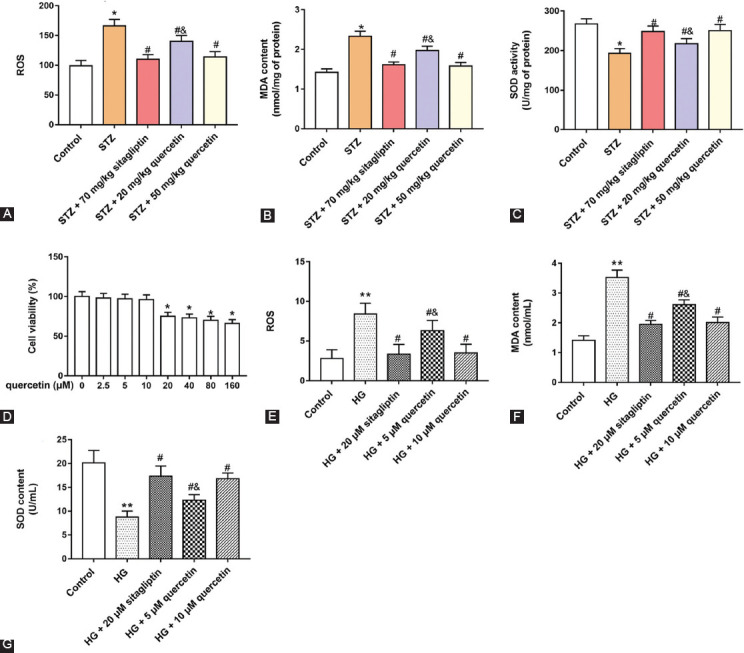
Effect of quercetin on oxidative stress indexes of testicular tissues and Leydig cells in rats. 2’-7’-Dichlorodihydrofluorescein diacetate assay was used to detect reactive oxygen species (ROS) production in testis tissue of rats (A). Enzyme-linked immunosorbent assay (ELISA) kits were used to detect the oxidative stress indexes, including malondialdehyde (MDA) content (B) and superoxide dismutase (SOD) activity (C) in the testis tissue of rats. Cells were treated with different doses (0, 2.5, 5, 10, 20, 40, 80 and 100 μM) of quercetin, and cell viability was detected by Cell Counting Kit-8 assay. (D) ROS production (E), MDA content (F) and SOD content (G) in cells were measured by 2’-7’-Dichlorodihydrofluorescein diacetate assay or ELISA. **P < 0.01 or *P < 0.05, compared with control group; ^#^*P <* 0.05, compared with Streptozotocin (STZ) or HG group; ^&^*P <* 0.01, compared with STZ + 70 mg/kg sitagliptin, STZ + 50 mg/kg quercetin group, HG + 20 μM sitagliptin or HG + 50 μM quercetin group.

To investigate the effect of quercetin on oxidative stress in diabetic rats, we examined ROS production, MDA content and SOD activity in rat testicular tissues and Leydig cells. Results of ELISA demonstrated that, *in vivo* or *in vitro*, STZ-induced rats increased ROS production and MDA content, and decreased SOD activity, compared with control group (*p* < 0.05). Administration of quercetin, especially, 50 mg/kg quercetin, decreased ROS production and MDA content, and increased SOD activity, and quercetin decreased ROS production (Figure 2A-C). Cells were treated with 0, 2.5, 5, 10, 20, 40, 80, and 160 μM quercetin, and 20, 40, 80, and 160 μM quercetin decreased cell viability (*p* < 0.05) (Figure 2D). *In vitro*, we also found that ROS production and MDA content were increased in HG-treated cells, and SOD content was decreased. Cells treated with quercetin decreased ROS production and MDA content, and increased SOD content (*p* < 0.05) (Figure 2E-G). These results confirmed that quercetin could reduce the oxidative stress in diabetic rats and HG-treated Leydig cells.

### Quercetin upregulated the expression of molecular biological indicators of testis in HG-treated Leydig cells

**FIGURE 3:**
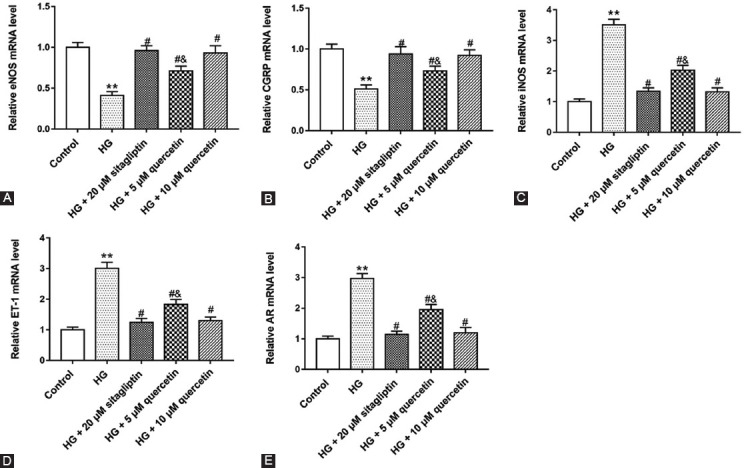
Effects of quercetin on the molecular biological indexes of HG-treated Leydig cells. Cells were treated with HG, 20 μΜ sitagliptin, 5 μΜ quercetin and 10 μΜ quercetin. Quantitative real-time polymerase chain reaction was used to detect the messenger RNA levels of eNOS (A), calcitonin gene-related peptide (B), iNOS (C), ET-1 (D) and AR (E) in cells. **P < 0.01, compared with control group; ^#^*P <* 0.05, compared with HG group; ^&^*P <* 0.01, compared with HG + 20 μΜ sitagliptin or HG + 10 μΜ quercetin group.

Considering testosterone secretion was reduced in STZ-induced rats, we studied the molecular biological indicators (eNOS, CGRP, iNOS, ET-1, and AR) of testis in diabetic rats. The messenger RNA (mRNA) levels of eNOS and CGRP were downregulated in HG-treated cells and upregulated in cells treated with two doses of quercetin (Figure 3A and B). Quercetin could eliminate the upregulation of iNOS, ET-1, and AR mRNA levels in HG-treated cells (Figure 3C-E). These results showed that quercetin could improve the hindered testosterone synthesis in HG-treated cells.

### Quercetin improved testosterone synthesis in STZ-induced rats and HG-treated Leydig cells

**FIGURE 4:**
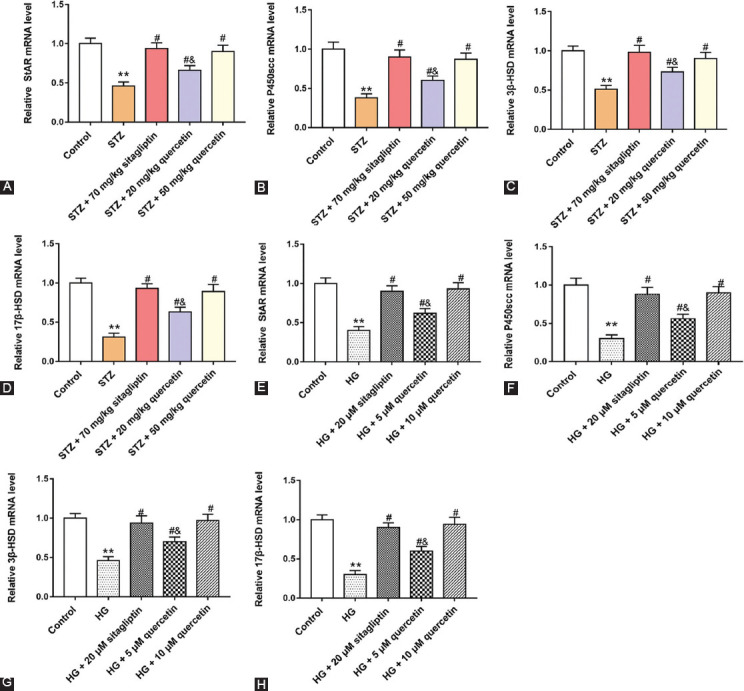
Effect of quercetin on messenger RNA (mRNA) levels of genes related to testosterone synthesis in Streptozotocin (STZ)-induced rats and HG-treated Leydig cells. The mRNA levels of StAR (A and E), P450scc (B and F), 3β-HSD (C and G) and 17β-HSD (D and H) were measured with Quantitative real-time polymerase chain reaction in rat testicular tissues and cells. **P < 0.01, compared with control group; ^#^*P <* 0.05, compared with STZ or HG group; ^&^*P <* 0.01, compared with STZ + 70 mg/kg sitagliptin, STZ + 50 mg/kg quercetin, HG + 20 μM sitagliptin or HG + 10 μM quercetin group.

To investigate the effect of quercetin on testosterone synthesis, we detected the mRNA levels of StAR, P450scc, 3β-HSD, and 17β-HSD. As shown in Figure 4A-D, STZ-induced rats decreased StAR, P450scc, 3β-HSD, and17β-HSD mRNA levels, and quercetin increased StAR, P450scc, 3β-HSD, and17β-HSD mRNA levels in testicular tissues. In addition, we found that quercetin reversed the decrease of StAR, P450scc, 3β-HSD, and 17β-HSD mRNA levels in HG-treated cells (Figure 4E-H). These results suggested that diabetes blocked the synthesis of testosterone in rats, and quercetin could help to improve this symptom.

### Quercetin relieved ER stress in STZ-induced rats and HG-treated Leydig cells

**FIGURE 5:**
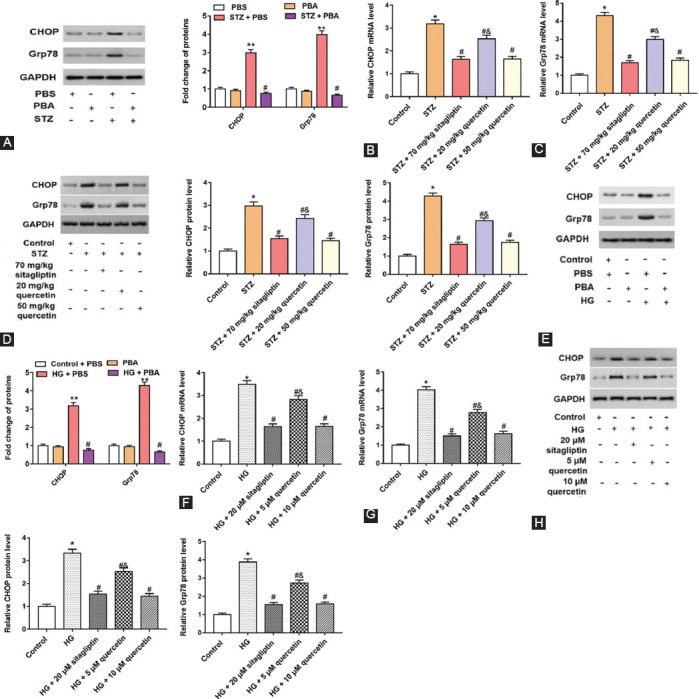
Effects of quercetin on the expression of endoplasmic reticulum (ER) stress-related proteins in testicular tissues and Leydig cells of rats. Streptozotocin (STZ)-reduced rats and HG-treated cells were treated by phosphate buffer saline (PBS) and Phenylbutyric acid (PBA). Western blot assay was performed to detect ER stress marker protein CHOP and Grp78 levels in STZ-induced rats (A) and HG-treated cells (E). **P < 0.01, compared with PBS group or control + PBS group; ^#^*P <* 0.05, compared with the PBA group. Quantitative real-time polymerase chain reaction and Western blot assay were used to measure the messenger RNA and protein levels of CHOP and Grp78 in testicular tissues (B-D) and cells (F-H). **P < 0.01, compared with control group; ^#^*P <* 0.05, compared with STZ or HG group; ^&^*P <* 0.01, compared with STZ + 70 mg/kg sitagliptin, STZ + 50 mg/kg quercetin, HG + 20 μΜ sitagliptin or HG + 10 μΜ quercetin group.

A previous study has shown that ER stress mediates glyphosate induced inhibition of testosterone synthesis in TM3 cells [23]. CHOP and Grp78 were used as markers of ER stress [24]. To study the mechanism of quercetin improving testosterone secretion, the protein levels of ER stress biomarkers (CHOP and Grp78) were detected in rat testicular tissues and Leydig cells. Phenylbutyric acid (PBA), an ER stress inhibitor [25], was used to inhibit the STZ-induced or HG-induced ER stress. STZ-induced rats increased CHOP and Grp78 protein levels, and PBA inhibited the STZ-induced ER stress in testicular tissues (Figure 5A). CHOP and Grp78 mRNA and protein levels were upregulated in rats administered with STZ compared with control rats, and two doses (20 and 50 mg/kg) of quercetin downregulated these levels (Figure 5B-D). *In vitro*, CHOP and Grp78 protein levels were also upregulated in HG-treated cells, compared with cells treated with PBS, and PBA treatment downregulated CHOP and Grp78 protein levels (Figure 5E). HG treatment increased CHOP and Grp78 mRNA and protein levels in HG-treated cells, and two doses (5 or 10 μM) of quercetin all decreased these levels (Figure 5F-H). These results suggested that quercetin could alleviate the stress of ER and regulate the disorder of testosterone secretion.

### MiR-1306-5p was downregulated in STZ-induced rats, and HSD17B7 was a target of miR-1306-5p

**FIGURE 6:**
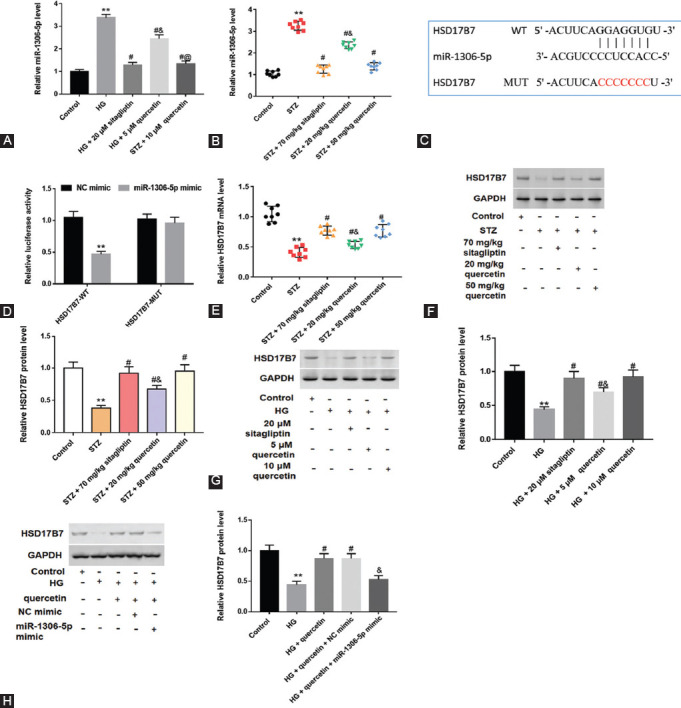
Effect of quercetin on expression of miR-1306-5p and HSD17B7 in rat testicular tissues and testicular Leydig cells. MiR-1306-5p level was determined with Quantitative real-time polymerase chain reaction (RT-qPCR) in rat testicular tissues (A) and cells (B). Starbase (http://starbase.sysu.edu.cn/index.php) (C) and Luciferase reporter gene assay (D) were used to predict and verify the binding between miR-1306-5p and HSD17B7. RT-qPCR and Western blot assays were conducted to measure the messenger RNA and protein level of HSD17B7 in testicular tissues (E and F) and cells (G). Cells were treated with HG and quercetin, then NC mimic and miR-1306-5p mimic were transfected into testicular Leydig cells. Cells were transfected with miR-1306-5p mimic or NC mimic (a negative control of miR-1306-5p mimic), and then treated with HG alone or together with quercetin: (H) Western blot assay was performed to determine HSD17B7 protein level in cells. **P < 0.01, compared with control group; ^#^*P <* 0.05, compared with Streptozotocin (STZ) or HG group; ^&^*P <* 0.01, compared with STZ + 70 mg/kg sitagliptin, STZ + 50 mg/kg quercetin, HG + 20 μΜ sitagliptin, HG + 10 μΜ quercetin, or HG + quercetin + NC mimic group.

As shown in Figure 6A and B, miR-1306-5p level was upregulated in testicular tissues of STZ-induced rats and HG-treated Leydig cells, and two doses of quercetin downregulated miR-1306-5p level. Starbase and Luciferase reporter gene assay predicted and confirmed that HSD17B7 was a target of miR-1306-5p (Figure 6C and D). HSD17B7 mRNA and protein levels were downregulated in STZ-induced rats and HG-treated cells, and two doses (20 and 50 mg/kg) of quercetin reversed downregulation of HSD17B7 mRNA and protein levels (Figure 6E-G). Moreover, miR-1306-5p reversed the increase of HSD17B7 protein level when cells were treated with HG and quercetin (Figure 6H).

### HSD17B7 overexpression reversed the increase of CHOP and Grp78 protein levels and eIF2α phosphorylation level caused by miR-1306-5p overexpression in HG-treated Leydig cells and STZ-induced rats

**FIGURE 7:**
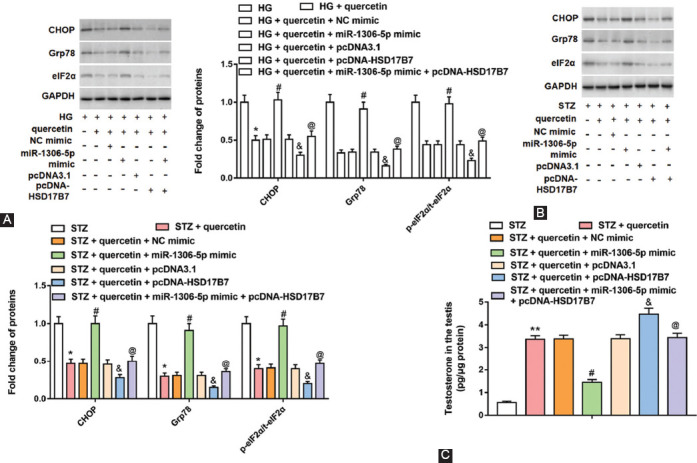
Effect of miR-1306-5p and HSD17B7 overexpression on endoplasmic reticulum stress related signaling pathway *in vivo* and *vitro*. Cells were transfected with miR-1306-5p mimic alone, NC mimic alone, pcDNA-HSD17B7 alone or together with miR-1306-5p mimic and pcDNA3.1 (a negative control of pcDNA-HSD17B7) alone. Streptozotocin (STZ)-induced rats were administered with quercetin alone or together with NC mimic, miR-1306-5p, pcDNA3.1, pcDNA-HSD17B7 or/and miR-1306-5p. Western blot assay was performed to detect the protein levels of CHOP and Grp78, and eIF2α phosphorylation level in testicular Leydig cells (A) and testicular tissues (B). (C) Testosterone secretion in the rat testis was detected with Enzyme-linked immunosorbent assay. **P < 0.01 or *P < 0.05, compared with HG group or STZ group; ^#^*P <* 0.05, compared with HG + quercetin + NC mimic group or STZ + quercetin + NC mimic group; ^&^*P <* 0.05, compared with HG + quercetin + pcDNA3.1 group or STZ + quercetin + pcDNA3.1 group; ^@^*P <* 0.05, compared with HG + quercetin + pcDNA-HSD17B7 group or STZ + quercetin + pcDNA-HSD17B7 group.

Leydig cells were transfected with miR-1306-5p mimic alone, NC mimic (a negative control of miR-1306-5p mimic) alone, pcDNA-HSD17B7 alone or together with miR-1306-5p mimic, and pcDNA3.1 (a negative control pcDNA-HSD17B7) alone. As shown in Figure 7A and B, quercetin decreased CHOP and Grp78 protein levels and phosphorylation level of eIF2α in HG-treated cells and STZ-induced rats, and miR-1306-5p reversed the downregulated of these protein levels. Overexpression of miR-1306-5p reversed the downregulation of CHOP, Grp78 and p-eIF2α levels caused by overexpression of HSD17B7. Then, we detected the testosterone in the testis and found that overexpression of HSD17B7 reversed the decrease of testosterone secretion caused by overexpression of miR-1306-5p (Figure 7C). Together, miR-1306-5p targeted HSD17B7 to induce the ER stress.

Overexpression of HSD17B7 reversed the increase of cell apoptosis caused by miR-1306-5p overexpression in HG-treated Leydig cells.

**FIGURE 8:**
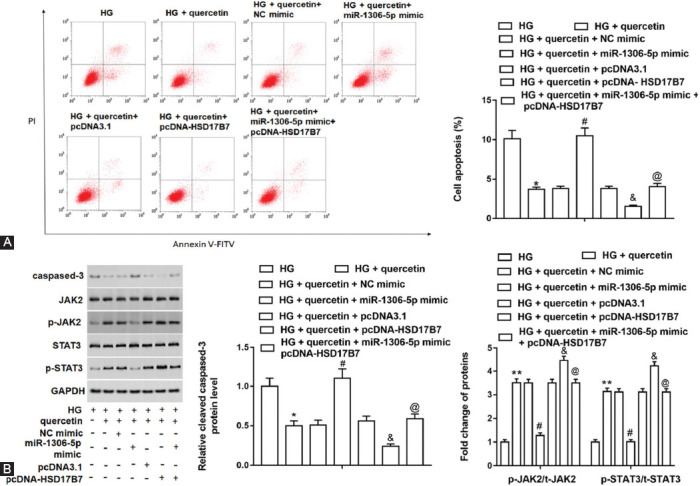
Effect of miR-1306-5p and HSD17B7 overexpression on cell apoptosis and the JAK2/STAT3 axis related protein levels. Cells were transfected with miR-1306-5p miamic alone, NC mimic alone, pcDNA-HSD17B7 alone or together with miR-1306-5p mimic and pcDNA3.1 alone. (A) Flow cytometry assay was performed to detect cell apoptosis. (B) Western blot assay was used to measure cleaved caspase-3 protein level and phosphorylation levels of JAK2 and STAT3 in testicular Leydig cells. **P < 0.01 or *P < 0.05, compared with HG group; ^#^*P <* 0.05, compared with HG + quercetin + NC mimic group; ^&^*P <* 0.05, compared with HG + quercetin + pcDNA3.1 group; ^@^*P <* 0.05, compared with HG + quercetin + pcDNA-HSD17B7 group.

Quercetin decreased cell apoptosis in HG-treated cells, and miR-1306-5p overexpression increased cell apoptosis. HSD17B7 overexpression reversed the facilitation of cell apoptosis caused by miR-1306-5p overexpression (Figure 8A). The JAK2/STAT3 signaling pathway had been reported in oxidative damage of testis in mice [35], and it was also reported in ER stress in rat heart ischemia/reperfusion injury [36]. MiR-1306-5p overexpression increased cleaved caspased-3 protein level and decreased JAK2 and STAT3 phosphorylation levels, compared with HG-induced cells treated with quercetin. HSD17B7 overexpression reversed the increase of cleaved caspased-3 level and the decrease of JAK2 and STAT3 phosphorylation levels caused by miR-1306-5p overexpression (Figure 8B). These results showed that miR-1306-5p targeted HSD17B7 to enhance cell apoptosis through the JAK2/STAT3 pathway.

## DISCUSSION

Quercetin is a natural polyphenol compound with anti-inflammatory [37], anti-oxidant [38], and blood sugar lowering properties [39]. The pharmacological effects of quercetin had been extensively studied in various research fields [40,41]. In this research, HG treatment increased the cleaved caspase-3 level and ER stress markers GRP78 and CHOP levels in Leydig cells. Quercetin significantly inhibited apoptosis-related protein levels and ER stress markers in cells treated by HG. In addition, quercetin could alleviate the inhibition of testosterone secretion induced by high glucose or STZ *in vitro* and *in vivo*.

The combination of 30 mg/kg quercetin and 10 mg/kg resveratrol played an important antidiabetic role in STZ-induced rats [42]. Moreover, 50 mg/kg quercetin could significantly reduce the morphological damage of liver cells in diabetic rats induced by STZ [32]. And in our research, administration of 50 mg/kg quercetin was better than 20 mg/kg in reducing blood sugar, oxidative stress, ER stress in STZ-induced testicular tissues. A study reported that compatibility with 5 μΜ quercetin might reduce the reproductive toxicity induced by Triptolide in rat Leydig cells [43]. 10 μΜ quercetin stimulated dcAMP-dependent activation of steroidogenic acute regulatory (Star), recombinant Cytochrome P450 11A1 (Cyp11a1) and ferredoxin-1 (Fdx1) promoters, which might increase the production of steroids in MA-10 Leydig cells [44]. In addition, we found that a testicular Leydig cell treated with 10 μΜ quercetin was better than 5 μΜ quercetin in reducing oxidative stress, ER stress and so on in HG-induced cells.

According to a report, in men with diabetes, it was often accompanied by a disorder of testosterone secretion [45]. The apoptotic rate of testicular cells was increased in diabetic rats [46]. To study the apoptosis mechanism induced by quercetin in Leydig cells, we first determined the effect of quercetin on the viability of Leydig cells. In this study, it was found that quercetin inhibited cleaved caspase-3 levels characterized by inhibition of Leydig cell apoptosis, which was consistent with one previous study [43]. The ER was the main organelle for the synthesis, maturation, and folding of proteins. To some extent, the fate of cells depends on the balance between survival and apoptosis signals, and specific ER stressors played a key role in regulating cell homeostasis [47]. A previous report showed that HG induced apoptosis of different types of cells by activating the ER stress-mediated apoptosis pathway [48]. CHOP proved to be a regulator of ER-related cell survival or death [49], and GRP78 was a protein folding partner that acted as a central regulator of ER stress response [50]. To confirm the role of ER stress in HG-induced interstitial cell apoptosis, we checked GRP78 and CHOP levels. Our data showed that GRP78 and CHOP protein levels were increased in quercetin treated Leydig cells. These results indicated that inhibiting ER stress in Leydig cells might effectively improve HG-induced imbalance of testosterone secretion.

MiRNAs are small non-coding RNAs that participate in gene transcription and post-transcriptional regulation by restricting the expression of target mRNA or causing mRNA degradation [51]. By affecting the stability of mRNA, they acted as negative regulators of protein translation, regulated numerous signal transduction pathways and cellular processes, and participated in cell-to-cell communication. MiR-1306-5p was downregulated in neuroblastoma cells (SH-SY5Y) induced by oxygen glucose deprivation by targeting Bcl2-interacting killer (BIK), but it was not largely reported in other diseases [52]. In this research, miR-1306-5p was upregulated in STZ-induced testicular tissues and HG-treated Leydig cells.

Activated STAT3 protein acted as a transcription factor to regulate cell proliferation, apoptosis, angiogenesis, tumor invasion and metastasis. JAK2/STAT3 is involved in the JAK/STAT family, an important signaling pathway involved in cell proliferation, apoptosis, immunity, and inflammation [53]. A previous study reported that Betulinic acid relieved T-2-Toxin-induced testicular damage and reduced the number of apoptotic cells, and significantly reduced the phosphorylation levels of JAK2 and STAT3 [35]. Di (2-ethylhexyl) phthalate, a environmental endocrine disruptor, caused significant changes in testicular morphology, decreased testicular organ coefficient, sperm count and the level of phosphorylation of STAT3 and increased testicular cell apoptosis and the level of oxidative stress [54]. Our results *in vitro* showed that quercetin treatment upregulated the phosphorylation levels of JAK2 and STAT3 in HG treated cells, revealing that the mechanism of testicular damage caused by different external factors may be inconsistent. In addition, a study revealed that the stress-associated ER protein 1 (Serp1) inhibited the apoptosis of rat cardiomyocytes H9c2 induced by hypoxia reoxygenation by inhibiting ER stress by activating the JAK2/STAT3 pathway [55]. It was reported that Fasudil protected the heart against ischemia-reperfusion injury by attenuating ER stress via activating the JAK2/STAT3 pathway [56], which suggested that activating of the JAK2/STAT3 pathway could inhibit ER stress. Our research also found that miR-1306-5p targeted HSD17B7 to induce ER stress and increase cell apoptosis by inhibiting the JAK2/STAT3 pathway *in vivo* and *in vitro*.

The limitation of the article may be that the sample size of rats is relatively small, and it is not very convincing to only use rats to make models without other mammals.

Quercetin inhibits ER stress through the miR-1306-5p/HSD17B7 axis and improves testosterone secretion disorders in diabetic rats.
